# The International Encyclopædia of Surgery

**Published:** 1885-02

**Authors:** 


THE INTERNATIONAL ENCLYCLOPÆDIA OF SURGERY. A systematic treatise on the Theory and Practice of Surgery. By Authors of Various Nations, edited by John Ashhurst, Jr., M. D., Professor of Clinical Surgery in the University of Pennsylvania. Illustrated with Chromo-lithographs and wood cuts, in six volumes. Vol. V. pp. 1207. William Wood & Company, New York: 1884.

This volume is fully equal to the preceding one of this Encyclopædia, as to the matter contained, and like all publications which come from this well known publishing house, is a perfect model of typographical neatness.

It begins with a paper by Professor Charles B. Nancrede, of the Philadelphia Polyclinic, which takes up the first one hundred and ten pages with "injuries of the head." Then follows "malformations and diseases of the head," by Frederick Freves, F. R. C. S., Assistant Surgeon to and Lecturer on Anatomy, at the London Hospital. This paper, though only occupying fifty-eight pages, is well written, and contains much that is new. Following this we have one hundred and twenty pages on injuries and dis ases of the eyes and their appendages, by E. Williams, M. D., Professor of Ophthalmology in the Miami Medical College. The lithographic plates, illustrative of the diseases discussed in this paper, are as fine as we ever saw.

Albert H. Buck, M. D., of New York, furnishes an excellent paper on "injuries and disease of the ear," which occupies seventy pages. This paper is well illustrated by chromo lithographs.

Ninety eight pages are taken up with a paper by George M. Lefferts, M. A., M. D., Clinical Professor of Laryngoscopy and diseases of the throat, in the College of Physicians and Surgeons of New York, on "diseases and injuries of the nose and its accessory sinuses."

Alfred C. Post, M. D., LL. D., emeritus Professor of Clinical Surgery in the University of the City of New York, contributes a paper on "injuries and disease of the face, cheeks and lips;" this is a most excellent addition to the literature of the subject and reflects great credit on the author.

"Injuries and diseases of the mouth, fauces, tongue, palate and jaws," by Christopher Heath, F. R. C. S., Holme Professor of Clinical Surgery in the University College, London, while a good paper, is not such as we would expect from the author with his well known reputation.

"Surgery of the Teeth and adjacent parts," has received proper attention at the hands of Norman W. Kinsley, M. D. S., D. D. S., late Professor of dental art and mechanism in the New York College of Dentistry.

In the following fifty-two pages George H. B. Macleod, M. D., F. R. C. S., and F. R. S. Edin. senior Surgeon to and lecturer on Clinical Surgery at the Western Infirmary; Regius Professor of Surgery in the University of Glasgow, Surgeon in ordinary to H. M. the Queen in Scotland, gives in a concise manner the latest known facts of "injuries and diseases of the neck."

"Injuries and diseases of the air passages," by J. Solis Cohen, M. D., Professor of Diseases of the Throat and Chest in the Philadelphia Polyclinic, is well written and is indeed a valuable paper.

"Injuries of the Chest," receives the proper attention at the hands of Edward H. Bennett, M. D., F. R. C. S. I., President of the Royal College of Surgeons in Ireland and Professor of Surgery in Trinity College, Dublin.

Thomas Annandale, F. R. C. S. E., Regius Professor of Clinical Surgery in the University of Edinburgh and senior Surgeon to the Edinburgh Royal Infirmary, discusses "diseases of the breast" in a masterly manner. This paper is well illustrated by life-like chromo-lithographs.

Henry Morris, M. A., and M. B. Lond. F. R. C. S., Eng., Surgeon to and Lecturer on Surgery at the Middlesex Hospital, London, occupies two hundred and sixty-two pages with "injuries and diseases of the abdomen," and presents much that is new in this department of surgery.

An excellent paper on "Hernia," by John Wood, F. R. S, F. R. C. S., Professor of Clinical Surgery in King's College, and Senior Surgeon to King's College Hospital, London, closes this most excellent volume.

The volume contains over three hundred well executed engravings and six full page chromo-lithographs, which are as near life-like as it is possible to have them. It is a valuable work alike to the specialist and general practitioner.

